# Effect of Supplemental Lutein and Zeaxanthin on Serum, Macular Pigmentation, and Visual Performance in Patients with Early Age-Related Macular Degeneration

**DOI:** 10.1155/2015/564738

**Published:** 2015-03-01

**Authors:** Yang-Mu Huang, Hong-Liang Dou, Fei-Fei Huang, Xian-Rong Xu, Zhi-Yong Zou, Xiao-Ming Lin

**Affiliations:** ^1^Peking University Health Science Center, 38 Xueyuan Road, Beijing 100191, China; ^2^Peking University Eye Center, Peking University Third Hospital, 49 Huayuan North Road, Beijing 100191, China

## Abstract

*Purpose*. To compare the 2-year effect of multiple doses of lutein/zeaxanthin on serum, macular pigmentation, and visual performance on patients with early age-related macular degeneration (AMD). *Methods*. In this randomized, double-blinded, and placebo-controlled trial, 112 early AMD patients randomly received either 10 mg lutein, 20 mg lutein, a combination of lutein (10 mg) and zeaxanthin (10 mg), or placebo daily for 2 years. Serum concentration of lutein/zeaxanthin, macular pigment optical density (MPOD), visual functions including best-spectacle corrected visual acuity (BCVA), contrast sensitivity (CS), flash recovery time (FRT), and vision-related quality of life (VFQ25) was quantified. *Results*. Serum lutein concentration and MPOD significantly increased in all the active treatment groups. Supplementation with 20 mg lutein was the most effective in increasing MPOD and CS at 3 cycles/degree for the first 48 weeks. However, they both significantly increased to the same peak value following supplementation with either 10 mg or 20 mg lutein during the intervention. No statistical changes of BCVA or FRT were observed during the trial. *Conclusions*. Long-term lutein supplementation could increase serum lutein concentration, MPOD, and visual sensitivities of early AMD patients. 10 mg lutein daily might be an advisable long-term dosage for early AMD treatment.

## 1. Introduction

Age-related macular degeneration (AMD) is the leading cause of irreversible vision loss among people aged over 50, especially in developed countries [1]. With the average population age increasing, the number of AMD patients is estimated to triple to 60–75 million worldwide in the next 30–40 years [2]. Since late AMD not only jeopardizes a patient's visual function and quality of life, but also brings a tremendous socioeconomic burden, most treatment strategies are focused on addressing late AMD [3, 4]. However, the treatment of AMD at an earlier stage might slow the progression before irreversible visual impairment occurs, which would be more effective in enhancing or maintaining visual performances [1, 5]. Unfortunately, no clinically proven therapies for early AMD exist at present, and few studies focus on early AMD [6, 7].

Substantial evidence suggested that lutein and its isomer zeaxanthin, also known as macular pigment (MP), might prevent the progression of AMD resulting from photooxidative damage [2, 8]. A meta-analysis showed that the dietary intake of lutein and zeaxanthin could lead to a 4% reduction in the risk of developing early AMD, as opposed to a 26% reduction for late AMD, indicating that lutein/zeaxanthin might be more effective in reducing the risk of progression from early AMD to late AMD [9].

Some intervention studies have shown putative functional benefits of lutein/zeaxanthin supplementation by increasing MPOD and visual functions; however, the evidence is limited and inconclusive [10–14]. Given the common recommendation to use long-term supplementation of lutein/zeaxanthin to treat AMD patients and the controversy around which dosage (20–40 mg/d versus 6–12 mg/d) is more effective [10, 15], it is surprising that only a few studies have specifically compared the long-term effects of different lutein/zeaxanthin dosages on early AMD without any interference from other nutrients. Moreover, although the amounts of zeaxanthin and lutein in the central 3 mm of macula are approximately the same, few researchers have studied the effect of using a supplementation where lutein and zeaxanthin are combined in equal doses [10, 11, 16]. Besides, few studies have observed the effects of lutein/zeaxanthin supplementation from multidimensions, including serum and macular concentrations, visual functions, and subjective evaluations.

Therefore, we conducted this 2-year randomized, placebo-controlled, double-blinded dose-ranging trial to determine the effects of lutein/zeaxanthin on serum concentration, MPOD, and visual performances in early AMD patients and to use the results to discuss the daily lutein/zeaxanthin dosage currently used for long-term treatment.

## 2. Methods

### 2.1. Subjects

Subjects with AMD aged over 50 years were recruited in Beijing, China. Inclusion criteria included a clinical diagnosis of early AMD (defined as the presence of soft drusen, presence of retinal pigmentary abnormalities with no signs of late AMD, or both) according to the Age-Related Eye Disease Study System [17], clear ocular media, and agreement to adhere to the study regimen. Those who had other ocular disorders or unstable systemic or chronic illness or consumed dietary supplements containing antioxidants or carotenoids within the previous 6 months were excluded. This study was performed in accordance with the principles of the Declaration of Helsinki and was approved by the Medical Ethics Committee of Peking University. Written consent was obtained from all subjects.

### 2.2. Study Design

All subjects were screened for eligibility based on the protocol criteria. Diagnosis of early AMD was confirmed by 2 ophthalmologists using funduscope and fundus photographs. After enrollment, subject information on characteristics and demographics was collected using questionnaires and examinations. Serum total cholesterol (TC), triglyceride (TG), high density lipoprotein-cholesterol (HDL-C), low density lipoprotein-cholesterol (LDL-C), and glucose were measured within 2 days of collection by Beijing Laweekse Health Laboratory using an autoanalyzer.

In this 2-year randomized, double-blinded, placebo-controlled trial, all subjects were randomly assigned to take either 10 mg lutein, 20 mg lutein, lutein (10 mg) + zeaxanthin (10 mg), or a placebo daily. All the supplements were packaged identically with the same labels. Serum lutein/zeaxanthin concentrations, MPOD, and visual performance indices including best-spectacle corrected visual acuity (BCVA), contrast sensitivity (CS), and flash recovery time (FRT) were quantified at baseline, 24 weeks, 48 weeks, and 2 years. All clinical examinations were performed by the qualified ophthalmic technicians in Peking University Eye Center, Peking University Third Hospital. Vision-related quality of life (VFQ-25) was measured at baseline, 48 weeks, and 2 years. Diet stability was assessed using a validated 120-item food frequency questionnaire conducted at baseline, 48 weeks, and 2 years. All subjects, examiners, and study staff were masked to treatment assignment.

Subjects were required to maintain their normal dietary and living habits and were asked to visit our office monthly to collect capsules of the following month and to return the remaining capsules from the month together with the daily checklist. They were encouraged to report any adverse effects immediately and were asked specifically about adverse events such as carotenoderma during visits.

### 2.3. Serum Lutein/Zeaxanthin

Serum concentrations of lutein and zeaxanthin were extracted and analyzed using a modified high-performance liquid chromatograph (HPLC) method, which is discussed in detail elsewhere [18]. The analysis was performed on a Hewlett-Packard/Agilent Technology Model 1100 HPLC System with a C30 column (5 *μ*m, 4.6 × 250 mm, Develosil, Japan) under the temperature of 25°C, detected at 450 nm. All procedures including the blood sample collections were performed under dim light.

### 2.4. MPOD

MPOD was determined using a confocal scanning laser ophthalmoscope (Heidelberg Retina Angiograph II, Heidelberg Engineering Inc., Heidelberg, Germany) and has been detailed elsewhere [19]. Argon laser light (488 nm) was used to excite autofluorescence (AF) after using infrared light, and a series of AF images were obtained for the excitation wavelengths quickly before recovery. All images were centered on the fovea and aligned to one average image according to their anatomic details. MPOD was quantified from this average image by comparing foveal and parafoveal AF. This measurement technique has been used in multiple clinical studies, and its accuracy and reliability were reported with a coefficient of variation of less than 5% [19–21].

### 2.5. Visual Performance

After diopter correction, BCVA was measured according to the Early Treatment Diabetic Retinopathy Study (ETDRS) protocol, and the results were converted to the logarithm of the minimum angle of resolution (logMAR) [22]. CS was measured with CSV-1000 test system (Vector-Vision, Dayton, OH) at 4 spatial frequencies (3, 6, 12, and 18 cycles/degree) with a grade scale from 1 (high contrast) to 8 (low contrast). The contrast level of the last correct response was defined as the CS of each frequency and is reported as log CS.

FRT was recorded using a macular adaptometer (MDD-2; Avenue Optical Flash LLC, Lighthouse Point, FL), which included a xenon arc, and flash filtered for infrared, ultraviolet, visible short wavelengths and was delivered through an aperture in a hand-held tube. Technicians pressed a push button to activate the flash and the timer and pressed it again to stop the timer when the first stimuli of vision recovery were reported by the subject. The time was recorded as FRT [23].

VFQ-25 was our primary patient-reported outcome to evaluate qualitative changes in visual function and health-related quality of life. Scores were calculated with a 0 to 100 scale where higher scores indicate better functioning [24]. The Chinese version of VFQ-25 used in this study has proven reliability and validity as a measure of vision-related functioning outcomes [25].

### 2.6. Sample Size and Statistical Analyses

Sample size estimations indicated that 26 patients per group were needed to be able to distinguish a 30% difference for MPOD change in treatment groups (5% significance level, power 80%), and a total of 112 patients were enrolled assuming a dropout rate of 10%. As for randomization, the sequence was computer generated in a 1 : 1 : 1 : 1 ratio within permuted blocks of size 8.

Baseline comparisons among groups were assessed using ANOVA or the chi-square analysis. Skewed data was logarithmically transformed for analysis. Differences between baseline and follow-up measurements within a group were assessed using paired *t*-tests, while between-group differences at each time point were tested using analysis of covariance. Changes between groups over time were assessed using repeated-measures ANOVA including a time × treatment interaction. The linear correlation between 2 variables was assessed using the Pearson test. Statistical analyses were conducted using SPSS 11.0 for Windows software (SPSS, Inc., Chicago, Illinois, USA). A 2-tailed *P* value of less than 0.05 was considered significant.

## 3. Results

### 3.1. Baseline Findings

Of the 334 screened participants, 112 subjects met all the criteria and were subsequently enrolled and randomized. Four subjects (3.6%) were excluded from the analysis due to their failure to attend scheduled examinations. Subject characteristics were well balanced across groups at baseline (Table 1). Of all subjects, 7 subjects (6.5%) were smokers and 7 (6.5%) were former smokers, with no differences among groups. Dietary intakes of lutein, zeaxanthin, beta-carotene, and other antioxidants were not significantly different among the groups or during the intervention (for all, *P* > 0.05). No adverse events related to the study were observed or reported. During the intervention, approximately 97% (105/108) of the subjects took at least 93% (missing 2 days) of their supplements every month.

### 3.2. Serum Lutein/Zeaxanthin Concentration and MPOD

**Figure 1:**
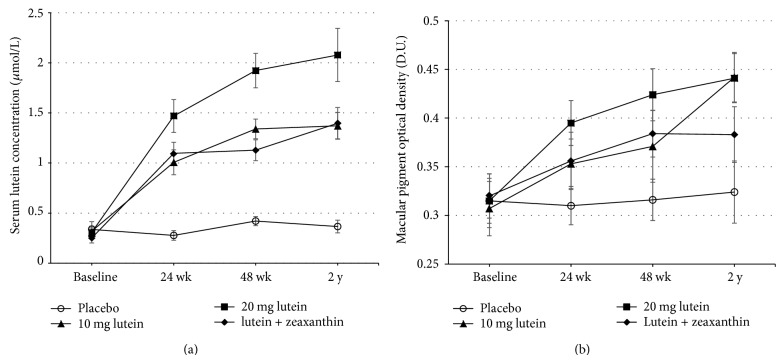
Changes in serum lutein concentration (a) and macular pigment optical density (b) at baseline, 24 weeks, 48 weeks, and 2 years in patients with early age-related macular degeneration, treated with 10 mg/d lutein, 20 mg/d lutein, lutein (10 mg/d) + zeaxanthin (10 mg/d), or placebo. Values are expressed as group mean ± SEMs. Significant increase was observed in all non-placebo groups compared to that of baseline or placebo group (all *P* < 0.05, paired *t*-test). Significant time and treatment effects were observed in serum lutein concentration, *P* < 0.001 (repeated-measures ANOVA), whereas only significant time effect was seen in MPOD, *P* < 0.001 (repeated-measures ANOVA).

Serum and macular concentrations of lutein/zeaxanthin in all the active treatment groups progressively increased (all *P* < 0.05), and the increases were all negatively correlated with their baseline values (all *P* < 0.001), whereas no such increases were seen in the placebo arm. Those who received 20 mg lutein showed a greater increase (6.75-fold) in serum lutein, compared to those who received 10 mg (4.30-fold) or lutein + zeaxanthin (5.57-fold) (Figure 1(a)). Serum zeaxanthin concentration significantly increased only in lutein + zeaxanthin group (3.87-fold, *P* < 0.001). The time × treatment interaction was significant for both serum lutein and zeaxanthin concentrations (both *P* < 0.001).

Likewise, the effect of 20 mg lutein on increasing MPOD was more effective at the first 24 weeks (increased by 25.4%, *P* < 0.01) and at 48 weeks (increased by 34.6%, *P* < 0.01). However, by year 2, the 10 mg lutein group reached the same MPOD level (0.442 D.U.) as the 20 mg lutein group (0.441 D.U.) (Figure 1(b)). Repeated-measures analyses showed a significant time × treatment interaction of MPOD (*P* = 0.046). MPOD significantly increased during the supplementation (*P* < 0.001), whereas no statistical treatment effect was shown (*P* = 0.072).

### 3.3. Visual Performance

Changes of CS among groups are shown in Table 2. By year 2, increments of CS at 3 and 6 cycles/degree from baseline were observed in all the active treatment groups, whereas a significant increase of CS at 18 cycles/degree was only seen in the lutein + zeaxanthin group. During the first 48 weeks, the increases of CS at 3 and 6 cycles/degree were higher and more significant (*P* < 0.01) after receiving 20 mg lutein. However, at 2 years, CS at 3 cycles/degree in the 10 mg lutein group significantly increased (+16.1%, *P* < 0.05) to a similar peak value to the 20 mg lutein group.  Table 3 showed that the effect of lutein/zeaxanthin supplementation on BCVA was not significant; however, significant differences of FRT compared to the placebo group were seen after receiving 10 mg lutein and 20 mg lutein at 2 years (*P* < 0.05). Repeated-measures analyses of the above variables did not reveal any differential treatment effects, except a significant time effect observed for CS at 3 cycles/degree (*P* < 0.05).

The VFQ25 scores did not show any significant change over the first 48 weeks; however, they slightly increased at 2 years, especially in the lutein + zeaxanthin group (increased by 7.9%, *P* < 0.01). Using a repeated-measures analysis of variance, the score increased significantly during the supplementation (*P* < 0.001), whereas no significant treatment effect was observed. The changes in VFQ25 scores from baseline to 2 years were correlated negatively with baseline scores in all active treatment groups (correlation coefficients from *r* = −0.40 to *r* = −0.60, all *P* < 0.05). Correlation analysis showed that baseline VFQ25 score was positively correlated with baseline BCVA (*r* = 0.24, *P* < 0.05) and CS at 4 spatial frequencies (correlation coefficients from *r* = 0.21 to *r* = 0.30, *P* < 0.05 for all).

## 4. Discussion

This trial demonstrated that 2 years of lutein/zeaxanthin supplementation increased serum lutein/zeaxanthin concentrations, MPOD, and visual performances in patients with early AMD, without leading to any detectable adverse effect. More interestingly, we found that though body lutein/zeaxanthin concentrations and visual performances increased the most after receiving 20 mg lutein within the first 48 weeks, the increases of MPOD and visual functions (BCVA and CS) were similar between the 10 mg lutein and the 20 mg lutein groups at 2 years. Additionally, our results indicate that a combined equal dose of lutein and zeaxanthin might be more effective in improving CS at 18 cycles/degree and patient-reported visual performance (VFQ25 scores).

Consistent with previous studies, lutein/zeaxanthin supplementation increased their serum concentrations dose-dependently [26]. Higher lutein or zeaxanthin dosage led to higher serum concentration, which is consistent with the straightforward kinetics of their transport in human blood [18]. However, although MPOD in the 20 mg lutein group was higher than that in other groups at 48 weeks, MPOD in the 10 mg lutein group steadily increased to a similar level to the 20 mg lutein group at 2 years. This indicated that the incorporation of lutein/zeaxanthin into the retinal tissue is not driven simply by diffusion [26] but is influenced by unique transport proteins in serum and binding proteins in human retina [27, 28]. The saturability of macular xanthophylls-binding protein may be responsible for the same MPOD level in the 10 mg and 20 mg lutein groups at 2 years [29]. This macular saturation of xanthophyll was also seen in the macula of rhesus monkeys [30]. Weigert's study also indirectly supports our notion, showing that MPOD higher than 0.5 D.U. was unlikely to increase during lutein supplementation [31]. Our study indicated that though higher lutein supplementation could rapidly increase its serum and macular concentration, a lower lutein dosage (10 mg/d) could reach and maintain an efficient MP level in the long term [11, 32].

In this study, a tendency of increase in BCVA and FRT was observed in all the active treatment groups (*P* > 0.01) [14, 33]. The latest Age-Related Eye Disease Study 2 (AREDS2) also showed nonsignificant increase of BCVA after 5 years of intervention using AREDS formulation (antioxidant vitamins C and E, beta carotene, and zinc) adding lutein (10 mg) + zeaxanthin (2 mg) and suggested that the inadequate dose and/or duration of treatment might be attributable to the lack of efficacy [34]. The insensitivity of BCVA and FRT tests might also be one explanation for the nonsignificant change. Significant morphologic changes usually do not adversely impact visual functions at an early stage, therefore leaving little room for measurable improvement [1, 5].

However, CS is a more sensitive visual indicator compared to BCVA and FRT, which could provide additional information at the very beginning of visual dysfunction. Significant increases of CS were indeed detected at different spatial frequencies at 48 weeks and 2 years in all the active treatment groups, which is in line with other studies [13, 35]. This might be due to the preferential absorption of MP on blue light (short-wave light that produces a veiling luminance), which would attenuate the adverse impact of chromatic aberration and improve visual function [36, 37]. We found that the increase patterns of CS at 3 cycles/degree after receiving 10 mg lutein and 20 mg lutein were in accordance with those of MPOD. However, unlike the significant increase of MPOD from baseline to 24 weeks, no statistical changes of CS were observed before 48 weeks. Our results indicated that MPOD might be the foundation for the improvements in visual functions; CS could only improve after MPOD had reached and maintained a relatively high level. This hypothesis is also supported by the positive correlation between changes in MPOD and improvements in visual functions as mentioned in other studies [38, 39].

We noticed that the change of MPOD and visual functions from baseline was smaller in this study compared to similar studies [13, 14]. One possible reason is that the baselines in our study were higher since the morphologic and functional macular impairments in early AMD did not seriously affect visual functions, and this may have left limited space for improvement. This in turn supports the notion that early intervention might be more effective and essential in enhancing or maintaining visual function. Though AREDS2 concluded that addition of lutein + zeaxanthin supplementation could not further reduce risk of progression to advanced AMD, we believe their results may not illustrate the actual effect of xanthophyll on AMD, since the supplemental lutein/zeaxanthin they used was combined with other antioxidants [34].

It should be noted that no significant improvement of VFQ25 score was observed until the second year, and the only significant change was seen in the lutein + zeaxanthin group. Similarly, in Richer's study, the VFQ25 score increased by only 2% after 12 months of lutein and/or zeaxanthin supplementation [10]. This is possibly because improved visual functions are the foundation for better vision-related quality of life and thus VFQ25 score could only increase after visual functions reached a certain level. Our notion was also supported by the positive correlation between VFQ25 scores and visual functions (BCVA and CS) detected in our study. Likewise, Revicki's study showed that mean VFQ25 scores correlated significantly with BCVA in eyes of AMD patients (*P* < 0.0001) [24].

There are some noteworthy limitations in this study. First, the high selective criteria for subjects may affect generalization. Second, since the progression of AMD from early stage to late stage is much longer than our intervention period, our study could not use late AMD as the ultimate outcome and, therefore, is not powered adequately to find a reduction in late AMD incidence. Larger-scale and longer-term studies should be undertaken to focus on the effects of lutein and/or zeaxanthin on early AMD, and more sensitive measurements should be used.

In conclusion, our study has shown that lutein/zeaxanthin supplementation could increase their serum concentrations, MPOD, and reverse visual impairment in subjects with early AMD. Most interestingly, our findings suggest that supplementation with either 10 mg or 20 mg lutein could be equally effective after 2 years. Thus, it might be advisable for early AMD patients to take a lower dosage (10 mg/d) for long-term treatment.

## Figures and Tables

**Table 1:** Baseline characteristics of subjects with early age-related macular degeneration^a,b^.

	Placebo (*n* = 28)	10 mg lutein (*n* = 26)	20 mg lutein (*n* = 27)	10 mg lutein + 10 mg zeaxanthin (*n* = 27)
Age (y)	69.0 ± 7.5	69.7 ± 8.3	69.3 ± 6.9	68.5 ± 6.9
Sex, male [*n* (%)]	11 (39.3)	9 (34.6)	14 (51.9)	12 (44.4)
Education (y)	12.2 ± 2.8	10.8 ± 2.7	12.2 ± 2.9	10.5 ± 4.1
BMI (kg/m^2^)	24.8 ± 3.0	24.1 ± 3.4	25.1 ± 3.3	24.6 ± 3.6
Serum lipids (mmol/L)				
Total cholesterol	5.022 ± 1.756	4.984 ± 1.068	5.091 ± 0.883	5.247 ± 0.952
Triglyceride	1.571 ± 1.575	1.538 ± 0.684	1.491 ± 0.821	1.777 ± 0.791
HDL cholesterol	1.388 ± 0.438	1.386 ± 0.319	1.408 ± 0.258	1.481 ± 0.291
LDL cholesterol	3.091 ± 0.606	3.190 ± 0.746	3.203 ± 0.605	3.338 ± 0.605
Early cataracts [*n* (%)]^c^	6 (21.4)	6 (23.0)	5 (18.5)	8 (29.6)
MPOD (D.U.)	0.315 ± 0.144	0.307 ± 0.142	0.315 ± 0.122	0.320 ± 0.118
Serum concentration (*μ*mol/L)				
Lutein	0.337 ± 0.397	0.319 ± 0.250	0.308 ± 0.231	0.251 ± 0.260
Zeaxanthin	0.066 ± 0.075	0.048 ± 0.050	0.050 ± 0.042	0.046 ± 0.055

^a^D.U.: density unit; MPOD: macular pigment optical density. There were no significant differences among groups in any of the baseline study characteristics noted.

^b^For continuous variables, all values are mean ± SDs. For categorical variables, all values are *n* values, percentages of the total in parentheses. Comparisons among groups were derived from analysis of variance for continuous variables or the chi-square test for categorical variables.

^c^Cataracts diagnosed and graded according to the Lens Opacities Classification System III.

**Table 2:** Changes of contrast sensitivity among different groups during the intervention^a^.

	Placebo (*n* = 28)	10 mg lutein (*n* = 26)	20 mg lutein (*n* = 27)	10 mg lutein + 10 mg zeaxanthin (*n* = 27)
Contrast sensitivity, log				
3 cycles/degree				
Baseline	1.22 ± 0.37	1.26 ± 0.36	1.24 ± 0.39	1.25 ± 0.32
24 weeks	1.22 ± 0.34	1.32 ± 0.39	1.34 ± 0.29	1.34 ± 0.34
48 weeks	1.13 ± 0.36	1.45 ± 0.37^†^	1.47 ± 0.39^∗∗†^	1.40 ± 0.31^*^
2 years	1.25 ± 0.32	1.47 ± 0.34^*^	1.32 ± 0.25^†^	1.39 ± 0.39^*^
6 cycles/degree				
Baseline	1.40 ± 0.39	1.41 ± 0.34	1.40 ± 0.39	1.45 ± 0.38
24 weeks	1.34 ± 0.34	1.47 ± 0.35	1.52 ± 0.37	1.51 ± 0.383
48 weeks	1.30 ± 0.31	1.57 ± 0.37	1.62 ± 0.36^***^	1.52 ± 0.38
2 years	1.25 ± 0.30	1.50 ± 0.33	1.54 ± 0.36^†^	1.50 ± 0.36
** **12 cycles/degree				
Baseline	0.97 ± 0.37	1.02 ± 0.33	1.00 ± 0.34	1.06 ± 0.36
24 weeks	1.02 ± 0.36	1.06 ± 0.42	1.06 ± 0.38	1.09 ± 0.35
48 weeks	0.91 ± 0.32	1.07 ± 0.35	1.12 ± 0.38	1.16 ± 0.40
2 years	0.87 ± 0.33	1.10 ± 0.35	1.05 ± 0.36	1.09 ± 0.35
18 cycles/degree				
Baseline	0.50 ± 0.35	0.57 ± 0.39	0.49 ± 0.35	0.53 ± 0.37
24 weeks	0.52 ± 0.36	0.60 ± 0.42	0.57 ± 0.38	0.63 ± 0.35
48 weeks	0.39 ± 0.28	0.62 ± 0.34	0.63 ± 0.38	0.68 ± 0.42
2 years	0.40 ± 0.34	0.59 ± 0.45	0.65 ± 0.39	0.74 ± 0.33^∗†^

All values are mean ± SDs. Mean values were significantly different from baseline within the same group: ^*^ *P* < 0.05,  ^**^ *P* < 0.01, and ^***^ *P* < 0.001. Mean values were significantly different from those of the placebo control group: ^†^ *P* < 0.05.

^a^Repeated-measures analyses of the above variables did not reveal any differential treatment effects, and the only significant time effect was observed at 3 cycles/degree (*P* < 0.05).

**Table 3:** Changes of visual performance among different groups during the intervention^a^.

	Placebo (*n* = 28)	10 mg lutein (*n* = 26)	20 mg lutein (*n* = 27)	10 mg lutein + 10 mg zeaxanthin (*n* = 27)
Best-corrected visual acuity, log⁡MAR				
Baseline	0.34 ± 0.19	0.31 ± 0.21	0.31 ± 0.21	0.32 ± 0.25
24 weeks	0.33 ± 0.25	0.32 ± 0.21	0.27 ± 0.17	0.28 ± 0.30
48 weeks	0.34 ± 0.22	0.28 ± 0.22	0.26 ± 0.20	0.27 ± 0.35
2 years	0.30 ± 0.25	0.26 ± 0.15	0.28 ± 0.16	0.27 ± 0.24
Photorecovery time, sec				
Baseline	18.57 ± 16.78	16.68 ± 14.22	15.86 ± 11.17	17.38 ± 12.00
24 weeks	19.02 ± 10.59	18.90 ± 17.71	14.13 ± 8.11	16.41 ± 14.69
48 weeks	19.70 ± 12.16	15.50 ± 11.27	14.61 ± 13.43	17.80 ± 16.48
2 years	24.41 ± 14.40	15.00 ± 8.40^†^	15.36 ± 12.75^†^	15.67 ± 11.04
VFQ25 score				
Baseline	76.04 ± 18.09	75.46 ± 14.60	75.58 ± 15.35	74.26 ± 14.46
48 weeks	74.97 ± 17.10	75.02 ± 13.01	72.56 ± 14.46	76.32 ± 11.20
2 years	77.31 ± 17.05	79.61 ± 13.52	76.65 ± 16.32	80.13 ± 11.73^**^

log⁡MAR: logarithm of minimum angle of resolution; VFQ25: Visual Function Questionnaire 25.

All values are mean ± SDs.

Mean values were significantly different from baseline within the same group: ^**^ *P* < 0.01.

Mean values were significantly different from those of the placebo control group: ^†^ *P* < 0.05.

^a^Repeated-measures analyses of the above variables did not reveal any differential treatment or time effects.
